# Case Report: Immune checkpoint inhibitor–induced hypophysitis presenting as acute paralytic ileus mimicking mechanical bowel obstruction in a patient affected by advanced non-small cell lung cancer

**DOI:** 10.3389/fonc.2026.1934901

**Published:** 2026-09-29

**Authors:** Alice Baggi, Anna Adriani, Luca Carlofrancesco Ammoni, Giorgia Carola, Filippo Morocutti, Lara Colombo Zefinetti, Simone Marzani, Alfredo Berruti, Salvatore Grisanti

**Affiliations:** 1 Medical Oncology, Comprehensive Cancer Center, Azienda Socio-Sanitaria Territoriale (ASST) Spedali Civili, Brescia, Italy; 2 Department of Medical and Surgical Specialties, Radiological Sciences and Public Health, University of Brescia, Brescia, Italy

**Keywords:** case report, hypophysitis, immune checkpoint inhibitors, intestinal pseudo-obstruction, non-small cell lung cancer

## Abstract

Immune checkpoint inhibitors (ICIs) have significantly improved outcomes in advanced non-small cell lung cancer (NSCLC) but may induce immune-related adverse events (irAEs), including hypophysitis. Although endocrine irAEs usually present with nonspecific constitutional symptoms, atypical manifestations may delay diagnosis and expose patients to unnecessary invasive procedures. We report the case of a 76-year-old man with metastatic NSCLC receiving first-line nivolumab, ipilimumab, carboplatin, and pemetrexed who developed profound asthenia, altered mental status, bilious vomiting, and severe gastrointestinal dilation suggestive of acute mechanical bowel obstruction after two treatment cycles. Computed tomography demonstrated marked gastric and intestinal distension, and surgical management was initially considered. Endocrine evaluation subsequently revealed central hypothyroidism and secondary adrenal insufficiency, consistent with ICI-induced hypophysitis. Prompt initiation of physiological hormone replacement with intravenous hydrocortisone followed by thyroid hormone supplementation resulted in rapid neurological and systemic recovery, complete restoration of gastrointestinal motility, radiological resolution of bowel dilatation, and avoidance of surgery. Follow-up imaging also demonstrated a partial tumor response, which has remained durable for more than two years without additional anticancer treatment while the patient continues long-term endocrine replacement therapy. This case expands the clinical spectrum of ICI-induced hypophysitis by describing an unusual presentation as acute paralytic ileus mimicking mechanical bowel obstruction. It underscores the importance of maintaining a high index of suspicion for endocrine irAEs in patients receiving ICIs who develop unexplained multisystem deterioration or apparent surgical abdomen. Early hormonal assessment and timely replacement therapy can be lifesaving, prevent unnecessary surgical interventions, and allow optimal multidisciplinary management without compromising oncologic outcomes.

## 1 Introduction

Immune checkpoint inhibitors (ICIs), through blockade of inhibitory immune pathways, such as programmed cell death-1 (PD-1) and cytotoxic T-lymphocyte-associated antigen-4 (CTLA-4), have substantially transformed the therapeutic landscape of advanced non-small cell lung cancer (NSCLC), leading to significant improvements in survival and long-term disease control compared with conventional chemotherapy and becoming a cornerstone of first-line treatment in metastatic disease (1).

However, ICI-induced immune system activation and response may also result in a distinct spectrum of toxicities known as immune-related adverse events (irAEs). These toxicities can involve virtually any organ system, reflecting a loss of immune tolerance and off-target inflammatory activation (2).

Endocrine irAEs are among the most frequent and clinically relevant complications, including thyroid dysfunction, hypophysitis, and, less commonly, primary adrenal insufficiency and diabetes mellitus (3).

While many endocrine irAEs are relatively manageable with hormone replacement therapy and temporary interruption/delay of immunotherapy, others may present with more atypical or severe clinical phenotypes requiring prompt recognition and multidisciplinary management. In particular, hypophysitis associated with ICIs can manifest with nonspecific and multisystem symptoms, potentially mimicking other acute conditions and leading to diagnostic delay (4).

Herein, we report the case of a patient with metastatic NSCLC treated with double-ICIs plus chemotherapy who developed an immune-related hypophysitis presenting with a severe gastrointestinal syndrome mimicking bowel obstruction.

## 2 Case description

A 76-year-old man was referred to our Oncology Department in December 2023, following surgical resection of a stenotic lesion of the distal ileum performed in another hospital. The procedure had been undertaken after the patient presented with symptoms of subacute intestinal obstruction. Histopathological examination of the resected specimen revealed metastatic adenocarcinoma with positive TTF-1 immunostaining consistent with a pulmonary primary oppure origin.

Baseline computed tomography (CT) imaging demonstrated a lesion in the right lower lobe of the lung, associated with multiple mediastinal and abdominal lymphadenopathies, as well as postoperative findings related to the recent ileal resection. Next Generation Sequencing (NGS) molecular profile did not identify any actionable genomic alterations, while programmed death-ligand 1 (PD-L1) expression was very low (tumour proportion score [TPS] 1%).

Apart from his oncological history, the patient’s past medical history was unremarkable and his family history was negative for relevant malignancies or known hereditary cancer syndromes.

Given the patient’s excellent clinical condition (Eastern Cooperative Oncology Group Performance Status [ECOG PS] 0), the absence of significant comorbidities, and the lack of chronic home medications, he was deemed as an appropriate candidate for first-line chemo- and double immunotherapy. Treatment with nivolumab, ipilimumab, carboplatin, and pemetrexed was therefore initiated in January 2024.

Approximately two weeks after the second cycle of chemo-immunotherapy, the patient presented at the Emergency Department with a marked deterioration in his general condition (ECOG PS 3–4). On examination, he appeared lethargic, poorly cooperative with space-time disorientation and disoriented to time and place. According to his family members, he had been experiencing progressive asthenia, anorexia, low-grade fever, and constipation over the preceding 4–5 days.

The patient was subsequently admitted to the Oncology Department with a working diagnosis of a possible infectious episode and sepsis despite the absence of neutropenia, given the elevated C-reactive protein (73 mg/L) and serum lactate level (3 mmol/L). Therefore, blood cultures were obtained and empiric broad-spectrum antibiotic therapy was initiated, together with intravenous hydration and parenteral nutritional support. Blood cultures later returned negative.

During hospitalization, the patient developed recurrent episodes of bilious vomiting which required placement of a nasogastric tube with significant improvement in abdominal distension. To investigate the possibility of an intra-abdominal infectious focus or disease progression, an abdominal computed CT scan was performed. Imaging revealed marked gastric and bowel dilatation, with a suspected mechanical obstruction secondary to an adhesional band (**Figure 1**).

**Figure 1:**
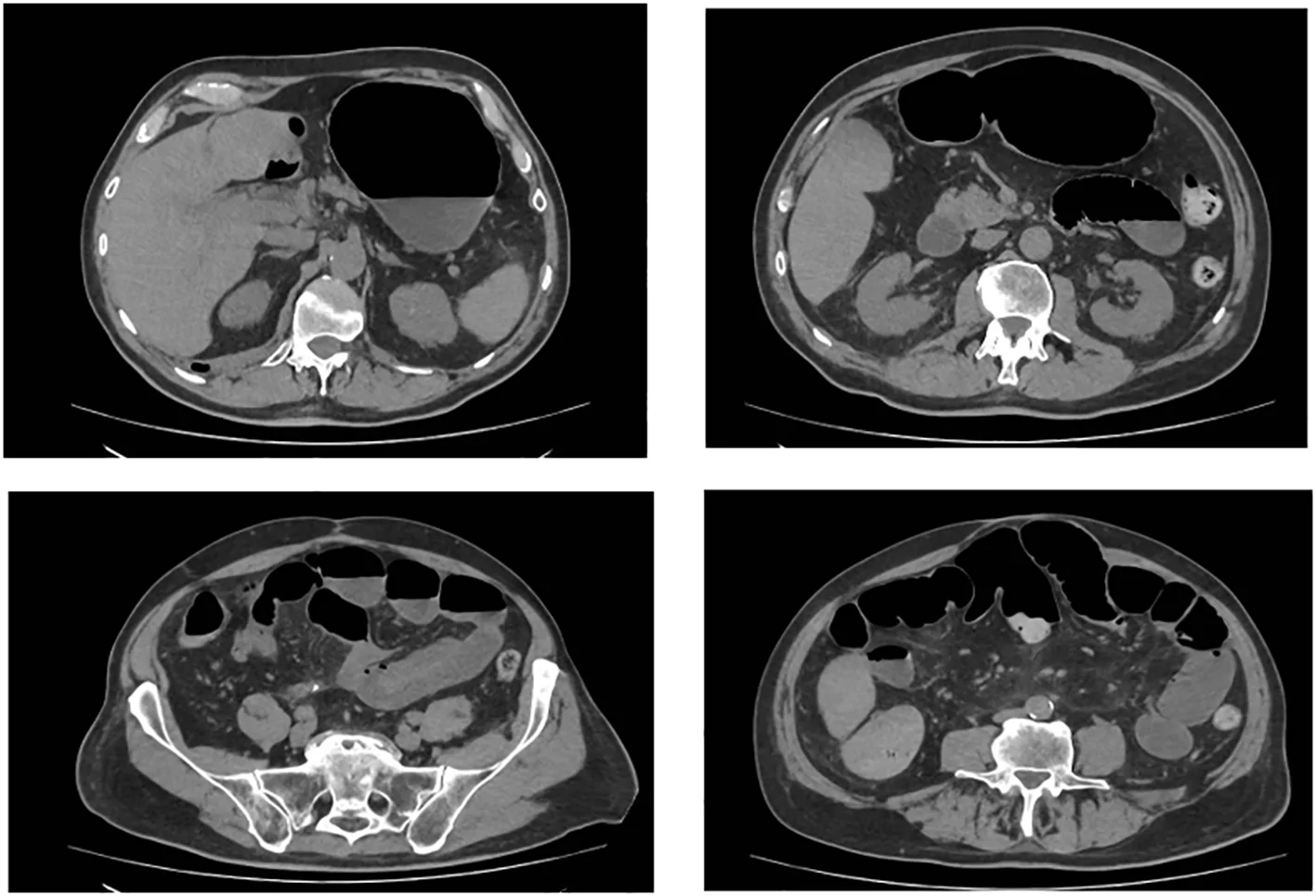
Computer tomography (CT) scan at hospital admission showing bowel obstruction signs.

A surgical consultation was therefore requested. While the suspected bowel obstruction was considered amenable to surgical correction and would ordinarily have warranted operative intervention, surgery was withheld owing to the patient’s severely compromised clinical condition. His poor performance status and frailty were judged to place him at excessively high risk for perioperative morbidity and mortality, leading to the decision to pursue conservative management.

Concurrently, laboratory testing showed central hypothyroidism, with low free thyroxine (fT4, 6.79 ng/L; reference range 9.3–17 ng/L) and free triiodothyronine (fT3, <1 ng/L; reference range 2–4.4 ng/L) levels associated with an inappropriately low thyroid-stimulating hormone (TSH, 0.223 mIU/L; reference range 0.270–4.2 mIU/L). In addition, secondary adrenal insufficiency was documented by reduced adrenocorticotropic hormone (ACTH, 5 ng/L; reference range 7–63 ng/L) and serum cortisol levels (3.9 μg/dL; reference range 4.8–19.5 μg/dL). The combined impairment of the thyrotropic and corticotropic axes was consistent with central hypothyroidism and secondary adrenal insufficiency, revealing evidence of anterior pituitary dysfunction and raising a strong clinical suspicion of immune checkpoint inhibitor-related hypophysitis.

Hormone replacement therapy was therefore promptly initiated with intravenous hydrocortisone (100 mg three times daily) and levothyroxine (25 μg/day). Following endocrinology consultation, hydrocortisone was continued at 100 mg three times daily for an additional 2–3 days and subsequently tapered to 50 mg four times daily. Levothyroxine was maintained at 25 μg/day, and liothyronine was added at a dose of 4 drops three times daily. Shortly after the introduction of hormonal supplementation, a marked clinical improvement was observed. The patient’s mental status progressively normalized, constitutional symptoms improved, and he gradually regained motor autonomy and the ability to perform activities of daily living. Notably, this clinical recovery was paralleled by a progressive resolution of the gastrointestinal manifestations, with restoration of bowel motility, disappearance of vomiting, and improvement of abdominal distension.

Although pituitary magnetic resonance imaging (MRI) was not performed and the remaining pituitary axes was not assessed, the temporal association with ICIs therapy and the marked clinical response to hormone replacement supported an immune-mediated pituitary disorder as the most likely contributor to the overall clinical picture, including the severe gastrointestinal manifestations.

An endocrinology reassessment was done after five days and led to an increase in levothyroxine to 50 μg/day, while liothyronine was maintained. Hydrocortisone was progressively tapered and subsequently switched to oral cortisone acetate, with gradual dose reduction according to clinical status and electrolyte balance. At follow-up 10 days later, levothyroxine was continued at 50 μg/day, liothyronine was discontinued, and cortisone acetate was maintained at 25 mg in the morning and 12.5 mg in the afternoon.

A follow-up CT scan subsequently confirmed the favorable evolution of the abdominal findings, demonstrating a marked reduction in gastric and intestinal dilatation and no evidence of persistent bowel obstruction. In view of the complete resolution of the obstructive-like picture and the patient’s clinical recovery, surgical intervention was no longer considered necessary, and oral feeding was successfully reintroduced. Given the patient’s previous ileal surgery and the initial response to nasogastric decompression, a transient or partial adhesive component cannot be completely excluded. Nevertheless the overall clinical and radiological course with the close temporal relationship between initiation of hormone replacement and progressive recovery of bowel motility, together with the subsequent resolution of gastric and intestinal dilatation without surgical intervention, supports a predominant endocrine contribution to the gastrointestinal syndrome and strongly suggests that pituitary hormone deficiency was the major driver of the severe intestinal dysmotility and pseudo-obstructive presentation.

Furthermore, imaging demonstrated a tumor partial response, with reduction of both right lower lobe mass and of the multiple mediastinal and abdominal lymphadenopathies.

The patient’s recovery continued over the following days, with progressive restoration of functional independence, and once a satisfactory level of autonomy had been achieved, he was discharged home with planned oncological and endocrinological follow-up.

From an oncological perspective, considering the sustained partial response observed after only two cycles of chemo-immunotherapy and the occurrence of a severe immune-related adverse event, no further systemic anticancer treatment was administered. The patient was therefore placed on active surveillance, with clinical assessment and CT imaging performed approximately every four months.

Remarkably, all subsequent radiological evaluations up to April 2026 have demonstrated ongoing disease control, with durable maintenance of the partial response and no evidence of disease progression. At the time of writing, the patient remains clinically stable on replacement therapy with levothyroxine 50 μg/day and cortisone acetate 25 mg in the morning and 12.5 mg in the afternoon, with endocrinological follow-up, and has experienced no recurrence of either endocrine or gastrointestinal symptoms. See **Figure 2** for the case timeline.

**Figure 2:**
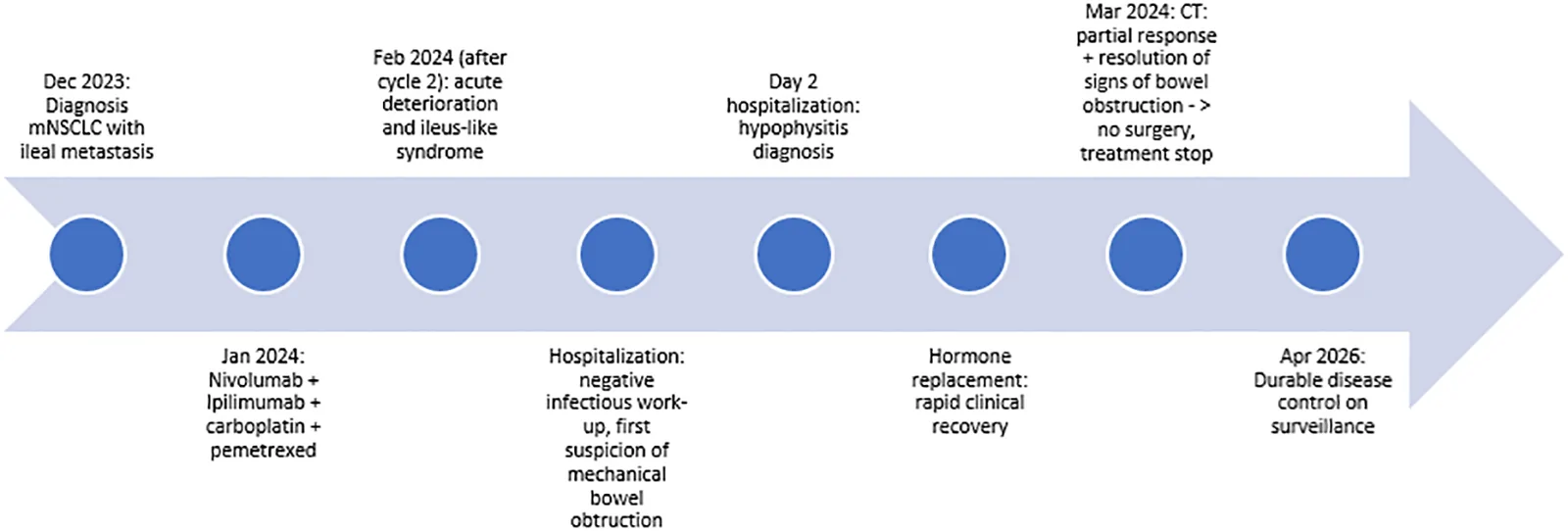
Patient’s case timeline (mNSCLC= metastatic non-small cell lung cancer).

## 3 Discussion

Hypophysitis is a well-recognized but relatively uncommon and potentially life-threatening immune-related Adverse Events (irAE), more frequently associated with CTLA-4 inhibitors than with PD-1/PD-L1 inhibitors monotherapy (5, 6). A meta-analysis of clinical trials reported a hypophysitis frequency of 6.4% with combination ICIs, 3.2% with CTLA-4 inhibitor monotherapy, and <1% with PD-1/PD-L1 inhibitor monotherapy, but observational studies have reported frequencies of up to 14% with CTLA-4 inhibitors and 0%-5% with PD-1/PD-L1 inhibitors (7, 8).

The median time of immune-related hypophysitis diagnosis has been reported to be around 8–10 weeks after immunotherapy initiation, often following the second or third dose of ICIs, and it is described more frequent in older, male patients (9). All these characteristics are confirmed in the presented case.

Immune-related hypophysitis may present with typical symptoms, such as headache and fatigue or visual disturbances (9) but it often manifests with subtle, non-specific features that can delay diagnosis.

In the present case, immune-related hypophysitis mimicked bowel obstruction, leading to an initial diagnostic and therapeutic approach focused on a surgical cause.

The gastrointestinal presentation deserves particular consideration because mechanical and functional causes may coexist, particularly in patients with previous abdominal surgery. In the present case, the severe gastrointestinal manifestations occurred in the context of profound corticotropic and thyrotropic dysfunction and improved markedly and progressively following initiation of glucocorticoid and thyroid hormone replacement. Importantly, this clinical improvement was accompanied by restoration of bowel motility and was subsequently confirmed by follow-up CT, which demonstrated a marked reduction in gastric and intestinal dilatation with no evidence of persistent mechanical obstruction. Moreover, the obstructive picture resolved without surgical intervention, despite the initial radiological suspicion of an adhesional band.

Although the patient’s previous ileal surgery and the initial CT findings indicate that a transient or partial adhesive component cannot be completely excluded, the overall clinical and radiological evolution strongly supports severe pituitary hormone deficiency as the predominant driver of the gastrointestinal syndrome. The close temporal relationship between endocrine replacement and resolution of both the systemic and gastrointestinal manifestations, together with the absence of persistent mechanical obstruction on follow-up imaging, provides compelling support for an endocrine-mediated intestinal dysmotility. We therefore hypothesize that immune checkpoint inhibitor-related hypophysitis, through severe secondary adrenal insufficiency and central hypothyroidism, was the principal contributor to the development and maintenance of the intestinal pseudo-obstructive presentation, which clinically mimicked a postoperative mechanical bowel obstruction. Adrenal insufficiency and severe hypothyroidism, in fact, are recognized causes of profound systemic manifestations, including hypotension, fatigue, neurocognitive impairment, but also gastrointestinal hypomotility and they may present with nausea, vomiting, abdominal distension, constipation, and, in severe cases, an intestinal pseudo-obstruction-like syndrome (9, 10).

The biological plausibility of an endocrine-mediated gastrointestinal dysmotility is supported by previous reports linking severe hypothyroidism, hypopituitarism, and adrenal insufficiency to intestinal ileus and pseudo-obstruction demonstrating that severe deficiency of pituitary-dependent hormones may impair gastrointestinal motility through combined metabolic and autonomic effects (11–13).

Our diagnosis was achieved only after endocrine assessment, emphasizing the need to consider endocrine immune-related adverse events in patients receiving immune checkpoint inhibitors with unexplained clinical deterioration.

Abnormalities in laboratory hormone levels are, in fact, the most common finding in cases of immune-mediated hypophysitis, with typical abnormalities indicative of hypocortisolism and hypothyroidism being observed in 60–100% of cases and of hypogonadotropic hypogonadism in 83–87% of male patients (14).

The finding of central hypothyroidism and secondary adrenal insufficiency led us to correctly contextualise the symptoms present and to diagnose an immune-related hypopituitarism.

A limitation of this case is the incomplete endocrine and radiological characterization of pituitary disfunction. The gonadotropic and somatitropic axes and prolactin secretion were not assessed, and pituitary MRI was not performed during the acute presentation, because both the thytotropic and corticotropic axes were clearly impaired, and immediate treatment was required. Therefore, the diagnosis of ICI-related hypophysitis was based on clinical and biochemical findings and the temporal relationship with nivolumab plus ipilimumab therapy rather than on radiological confirmation.

While biochemical evaluation represents the cornerstone of diagnosis, the role of pituitary MRI deserves consideration. Although pituitary MRI is recommended by current guidelines, particularly in patients with symptoms related to pituitary enlargement, normal imaging does not exclude immune-related hypophysitis (15, 16). In our patient, the absence of imaging and a complete pituitary hormonal profile limits the ability to definitively exclude other causes of pituitary disfunction. However, the rapid clinical response to hormone replacement further supported the diagnosis, highlighting that therapy should not be delayed while awaiting radiological confirmation when clinical suspicion is high.

Another important aspect of this case is its management. Our patient was successfully treated with physiological hormone replacement rather than high-dose glucocorticoids. This approach is supported by current evidence, including the randomized CORTICI trial (17), showing that high-dose corticosteroids do not improve pituitary hormonal recovery and may be associated with less favorable metabolic outcomes. Accordingly, hormone replacement alone is appropriate for most patients without compressive symptoms, whereas high-dose glucocorticoids should be reserved for severe presentations or pituitary mass-effect manifestations, as also emphasised in the guidelines (15, 16).

From an oncological perspective, our patient achieved a durable partial response after only two cycles of chemo-immunotherapy, which has been maintained without further systemic treatment.

Although immune-related adverse events, particularly endocrine toxicities, have been associated with improved survival in patients with advanced NSCLC in several studies (18, 19), this relationship remains controversial because of conflicting evidence and potential methodological biases (20). Data specifically addressing immune-related hypophysitis are even more limited and inconsistent possibly because its low incidence and the morbidity associated with this toxicity may obscure any potential survival benefit (8). Thus, while the durable response observed in our patient after the development of severe endocrine toxicity is consistent with previous reports, no causal relationship can be established. The main message of this case remains the importance of timely recognition and management of this potentially life-threatening endocrine toxicity.

## 4 Conclusion

This case highlights that immune-related hypophysitis should be considered in any patient receiving immune checkpoint inhibitors who develops unexplained clinical deterioration, even when the presentation is misleading and dominated by non-endocrine symptoms. Prompt endocrine assessment and timely hormone replacement are crucial to avoid unnecessary interventions, prevent life-threatening complications, and preserve the opportunity to achieve the full benefit of immunotherapy.

## Data Availability

The original contributions presented in the study are included in the article. Further inquiries can be directed to the corresponding author. Requests to access these datasets should be directed to Alice Baggi, a.baggi001@unibs.it.
